# Relationship between standardized uptake value on 18F-FDG PET and PD-L1 expression in clear cell renal cell carcinoma

**DOI:** 10.3389/fonc.2022.1012561

**Published:** 2022-10-04

**Authors:** Ying Liu, Yanlei Huo, Chao Ma, Zhongwei Lv

**Affiliations:** Department of Nuclear Medicine, Shanghai Tenth People’s Hospital, Tongji University School of Medicine, Shanghai, China

**Keywords:** clear cell renal cell carcinoma, PET/CT, lymph node metastasis, SUVmean, PD-L1

## Abstract

**Purpose:**

Partial clear cell renal cell carcinoma (CCRCC) may be sensitive to immune checkpoint inhibitor treatment targeting the programmed cell death 1 (PD-1)/programmed cell death 1 ligand 1 (PD-L1) pathway. Assessing the levels of PD-L1 using non-invasive imaging is useful to select immunotherapy-sensitive patients. Currently, whether PD-L1 levels in CCRCC correlate with 18F fluorodeoxyglucose (18F-FDG) uptake is unknown. This study aimed to assess whether 18F-FDG-positron emission tomography (PET) imaging could be used to infer PD-L1 levels in CCRCC.

**Methods:**

Immunohistochemistry (IHC) was used to assess PD-L1 levels in samples of tumors obtained retrospectively from a cohort of 58 patients with CCRCC who also received 18F-FDG PET/CT imaging. The IHC scores for PD-L1 were compared with the 18F-FDG maximum standardized uptake value (SUVmax), and the mean standardized uptake value (SUVmean) value, with the clinical characteristics of CCRCC, and with the IHC scores of enzymes related to glucose metabolism (glucose transporter type 1 (GLUT1), hexokinase 2 (HK2), lactate dehydrogenase A (LDHA)), and Von Hippel-Lindau tumor suppressor (VHL).

**Results:**

Increased renal venous invasion, lymph node metastasis, tumor size, SUVmean, and SUVmax correlated significantly with higher PD-L1 levels (P < 0.05). The IHC scores of VHL and LDHA correlated positively with those of PD-L1 (P = 0.035, P = 0.011, respectively). Significant correlations between PD-L1 levels and SUVmean and lymph node metastasis were observed upon multivariate analysis. SUVmean combined with lymph node metastasis predicted that 20.59% of the low probability group would express PD-L1, 29.41% of the medium probability group would express PD-L1, and 71.43% of the high probability group would express PD-L1.

**Conclusion:**

The status of lymph node metastasis, SUVmax, and SUVmean of the primary lesion correlated with PD-L1 levels in CCRCC. A combination of lymph node metastasis status and SUVmean could be utilized to predict PD-L1 levels, thus allowing monitoring of a tumor’s immunotherapy response.

## Introduction

The incidence of renal cancer has seen a worldwide increase in recent years, and renal cancer now represents the most lethal urinary system malignancy (1). The most common pathological type of renal cancer is clear cell renal cell carcinoma (CCRCC). CCRCC is often insensitive to chemotherapy and radiation therapy, and this insensitivity presents a substantial clinical challenge (2). Recently, immunotherapy has come to the forefront of clinical research. Programmed cell death 1 (PD-1) and programmed cell death ligand 1 (PD-L1) are closely related to cellular and humoral immunity (3). PD-1 is an immunosuppressive receptor. The ligands of PD-1 are PD-L1 and PD-L2; however, PD-L1 is the major ligand (4). Partial malignant tumors express PD-L1, including renal cell carcinoma (2), non-small cell lung cancer (5), and breast cancer (6). PD-1 and PD-L1 inhibitors have demonstrated efficacy in a variety of malignancies, including renal cancer (7).

Not all tumor cells express PD-L1; therefore, it is necessary to evaluate the levels of PD-L1 in tumor cells using immunohistochemistry (IHC) or other pathological methods before treating patients with targeted inhibitors of PD-L1. Although pathological diagnosis is the gold standard to assess PD-L1 levels, when many lesions are present, it is impossible to perform multiple biopsies to evaluate each individual lesion. Alternatively, the tumor phenotype might be predicted using noninvasive strategies, such as positron emission tomography (PET)/computed tomography (CT). Research has shown that PET/CT could potentially predict the tumor phenotype, and has been utilized to indicate PD-L1 status in lung cancer or head and neck cancer (8). However, there are no relevant studies reporting whether there is a correlation between PD-L1 levels and the standardized uptake value (SUV) of renal cancer.

Herein, 58 patients with CCRCC who underwent 18F-FDG PET/CT examination were analyzed. Five biological markers were evaluated: PD-L1, glucose transporter type 1 (GLUT1), hexokinase 2 (HK2), lactate dehydrogenase A (LDHA) and Von Hippel-Lindau tumor suppressor (VHL). We aimed to investigate the correlations between PD-L1 levels and selected tumor markers and the parameters of 18F fluorodeoxyglucose (18F-FDG)-PET/CT; and to determine whether CCRCC PD-L1 levels could be predicted using 18F-FDG-PET/CT.

## Materials and methods

### Patients

This study comprised a retrospective analysis. The cases studied include 58 patients with pathologically confirmed CCRCC who underwent 18F-FDG PET/CT scans at the Nuclear Medicine Department of Shanghai 10th People’s Hospital from July 2018 to June 2021. This cohort included 41 males and 17 females, aged 62 (range: 31–82) years old. The inclusion criteria were: (1) Pathologically confirmed CCRCC lesions; (2) no more than 2 weeks between surgical pathological confirmation and PET/CT scanning. The criteria outlined in the 7th edition of the American Joint Committee on Cancer (AJCC)/International Union against Cancer (ULCC) staging system were used to carry out tumor-node-metastasis (TNM) staging. These criteria were met by all 58 patients. In this study, informed consent was waived. The Institutional Review Board of Shanghai 10th Hospital approved the study, which was carried out following the tenets of the Declaration of Helsinki, as revised in 2013.

### ^18^F-FDG PET/CT examination

A Biograph 64 PET/CT device (Siemens, Munich, Germany) was used to examine the patients. Briefly, before the examination, the patients fasted for 4–6 h, such that their blood glucose decreased to below 6.3 mmol/L. Then, 18F-FDG (Shanghai Kexin Pharmaceutical Co., Ltd., Shanghai, China; radiochemical purity > 95%) at 5.55 MBq/kg body weight was injected intravenously. The patients then rested for 60 min and urinated before the PET/CT examination. In the supine position, the patients were scanned using the following parameters: CT scanning parameters: voltage: 120 kv, current: 140 mA; PET scanning parameters: three-dimensional model, 2 min/bed, scanning bed height adjusted according to the patient’s height (normal adult: 6), matrix: 128 * 128. Attenuation correction of the PET images with CT was carried out after collection and ordered subset expectation maximization (OSEM; 2 iterations, 28 subsets) reconstruction was used to obtain the PET tomographic images. A Siemens post-processing workstation produced the PET/CT fusion images.

### Analysis of imaging data

Two experienced nuclear medicine physicians read all the images. We exported all the data to the Nebula workstation (IntelliSpace Portal v7.0; Philips; Amsterdam, Netherlands). The TUMOR TRACE software automatically outlined the lesion boundary, followed by automatic calculation of SUVmax and SUVmean. A nuclear medicine physician checked the automatically calculated results.

### Immunohistochemical staining

Biopsies samples taken from resected tumors were stored in liquid nitrogen at −80°C. IHC staining was performed using 5 µm thick formalin-fixed paraffin sections. Staining was accomplished using antibodies recognizing GLUT1, HK2, LDHA,VHL, and PD-L1. Abcam (Cambridge, MA, USA) provided all the primary antibodies. Two board-certified pathologists evaluated the IHC results. The positive staining intensity was determined by light microscopy as follows: colorless - (counted as 0); faint yellow as + (counted as 1); brownish yellow as ++ (counted as 2); brown as +++ (counted as 3). For the positive staining area, a percentage value ≤ 5 was considered negative (counts as 0); 5 < the positive area percentage value ≤ 10 was considered + (counted as 1); 10 < the positive area percentage value ≤ 20 was considered ++ (counted as 2); and a positive area percentage value > 20 was considered +++ (counted as 3). The total staining score was determined by multiplying the percent positive intensity by the positive area; a total staining score < 4 was considered as low expression. A total staining score ≥ 4 was considered as high expression (9).

### Statistical analysis

Data were analyzed using SPSS 16.0 software (IBM Corp., Armonk, NY, USA). Data are presented as the mean ± standard deviation (x ± s). For differences in means measured using an independent-sample t-test, P < 0.05 was considered statistically significant.

## Results

### Correlation of the PD-L1 level with clinicopathological characteristics in patients with CCRCC

**Figure 1:**
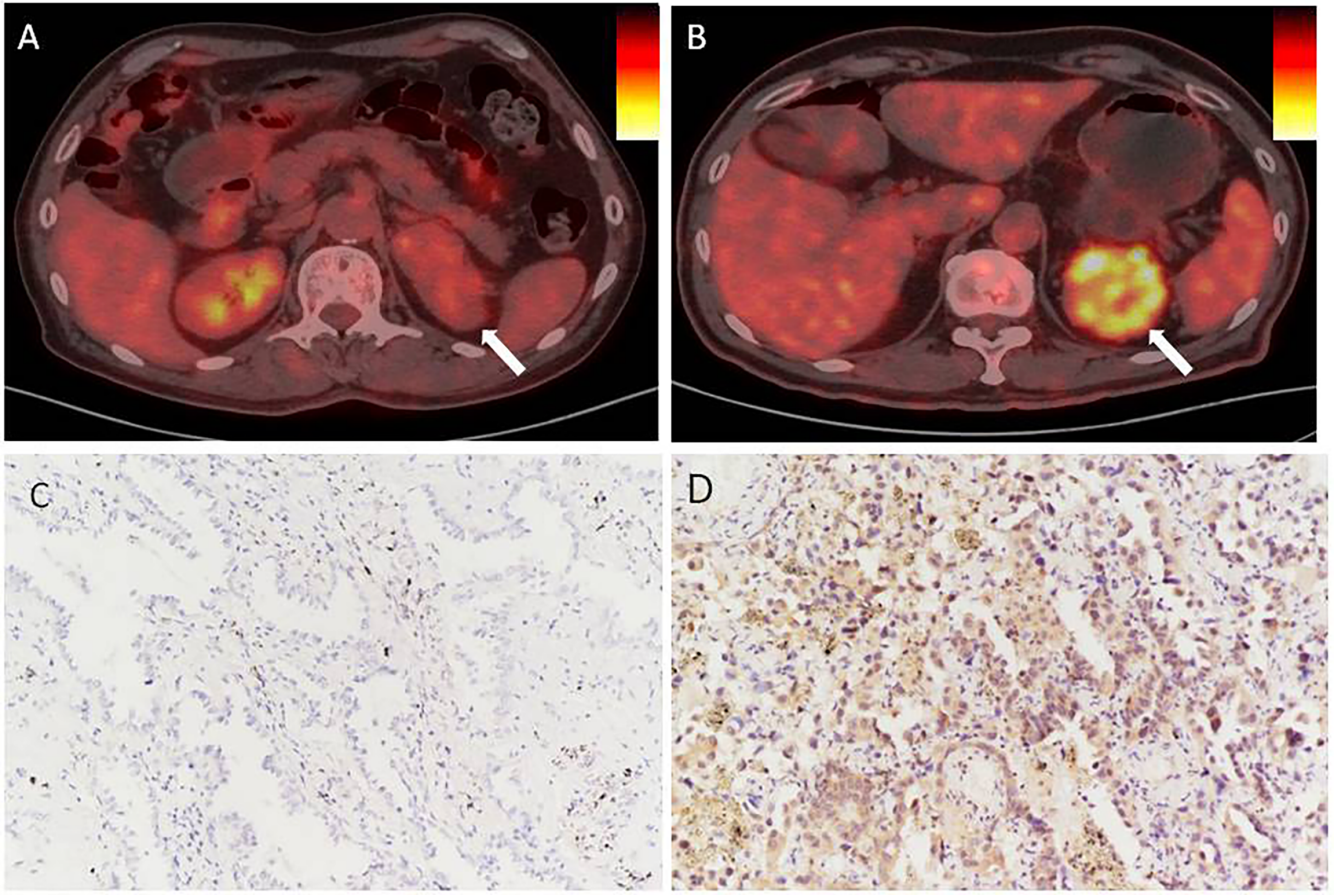
In renal cancer, there is an association between PD-L1 levels and SUVmax. **(A, C)** Negative staining for PD-L1 in a tumor from a 66 year man with CCRCC. The tumor lesion did not take up 18F-FDG obviously (SUVmax = 3.4, SUVmean = 1.9). **(B, D)** Strong positive staining for PD-L1 in a tumor from a 71 year old woman with CCRCC. The 18F-FDG PET/CT scan showed obvious accumulation of 18F-FDG in the tumor (SUVmax = 8.3, SUVmean = 3.5). IHC images were captured at 200× magnification.

First, PD-L1 levels in primary CCRCC lesions were analyzed using IHC. We evaluated the relationship between PD-L1 levels and patient gender, tumor size, age, degree of differentiation, renal vein tumor thrombus, lymph nodes, and distant metastasis, and SUVmax and SUVmean of primary lesions. PD-L1 levels were not significantly associated with patient gender, age, or distant metastasis. In addition, there were no significant differences in PD-L1 levels between well-differentiated and poorly differentiated tumors. However, PD-L1 levels were significantly associated with renal venous invasion, tumor size, and lymph node metastasis (**Table 1**). In particular, SUVmax and SUVmean values were significantly higher in primary tumors with high PD-L1 levels than in those with low PD-L1 levels (P <0.05) (**Table 1**). **Figure 1** shows typical images of PD-L1 IHC staining and 18F-FDG PET/CT scans of tumors with positive or negative PD-L1 expression.

**Table 1:** Relationship between PD-L1 expression and clinical characteristics.

Variable	n	PD-L1 expression		*P*-value
		Low	High	
**Age**				
< 55	16	14	2	0.083
≥ 55	42	27	15	
**Gender**				
Female	17	11	6	0.519
Male	41	30	11	
**Tumor size (cm)**				
≤ 4	23	20	3	0.027
> 4	35	21	14	
**Lymph node metastasis**				
Negative	47	36	10	0.041
Positive	11	5	7	
**Renal venous invasion**				
Negative	50	39	11	0.002
Positive	8	2	6	
**Distant metastasis**				
Negative	47	33	14	0.869
Positive	11	8	3	
**Tumor differentiation**				
Well-differentiated	40	31	9	0.089
Poorly differentiated	18	10	8	
**Tumor necrosis**				0.819
No	52	37	15	
Yes	6	4	2	
**SUVmax**		4.568 ± 4.220	7.071 ± 5.156	0.040
**SUVmean**		2.983 ± 2.234	4.553 ± 2.847	0.032

### Correlation between PD-L1 expression and SUVmax and SUVmean in renal cancer

**Figure 2:**
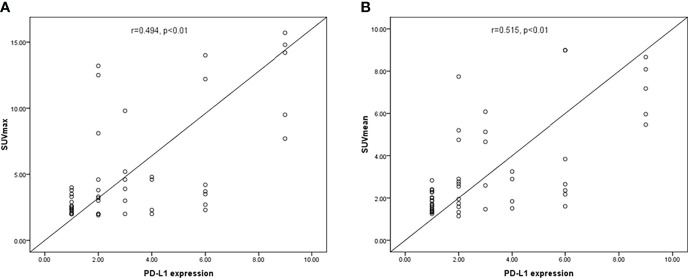
The correlation between PD-L1 levels and SUVmax and SUVmean in CCRCC. **(A)** SUVmax correlated positively with PD-L1 levels (P < 0.01). **(B)** A significant positive correlation was found between PD-L1 levels and SUVmean (p < 0.01).

**Figure 3:**
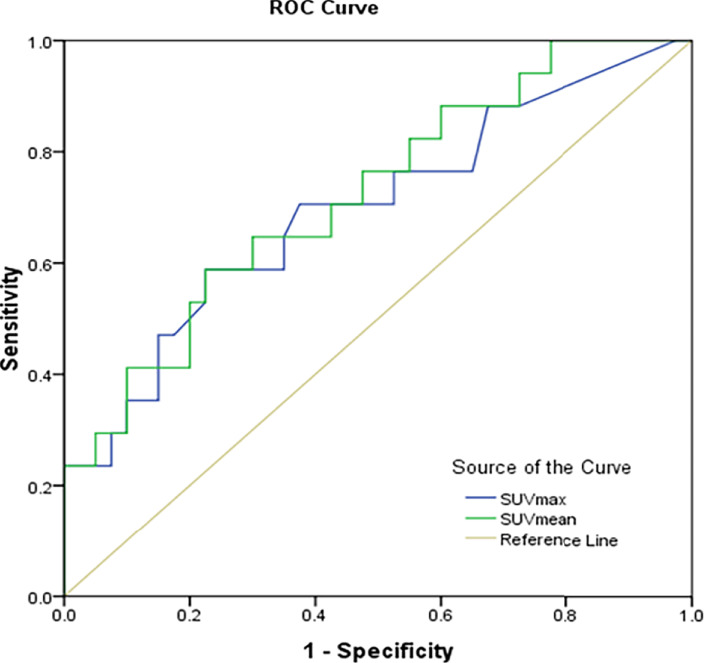
Prediction of PD-L1 expression in CCRCC using receiver operator characteristic curve analysis (ROC) of SUVmax and SUVmean.

The SUVmax and SUVmean values were significantly different between renal cancer lesions with high and low levels of PD-L1. Therefore, the correlation PD-L1 levels and tumor SUVmax and SUVmean in renal cancer were analyzed, which showed that tumor SUVmax and PD-L1 levels correlated positively, with a correlation coefficient (r) of 0.494 (P < 0.01; **Figure 2A**). Similarly, tumor SUVmean and PD-L1 levels correlated positively (r = 0.515, p < 0.01) (**Figure 2B**). Next, receiver operating characteristic (ROC) curves were used to determine if SUVmax or SUVmean values could predict PD-L1 levels. Analysis of the curve showed that SUVmax and SUVmean could be used to predict PD-L1 levels; the areas under the curve were 0.696 ± 0.080 and 0.722 ± 0.074 (**Figure 3**), with optimal cut-off values of 4.1 and 2.9, respectively.

### Correlation between PD-L1 levels and enzymes related to glucose metabolism and VHL in renal cancer

**Figure 4:**
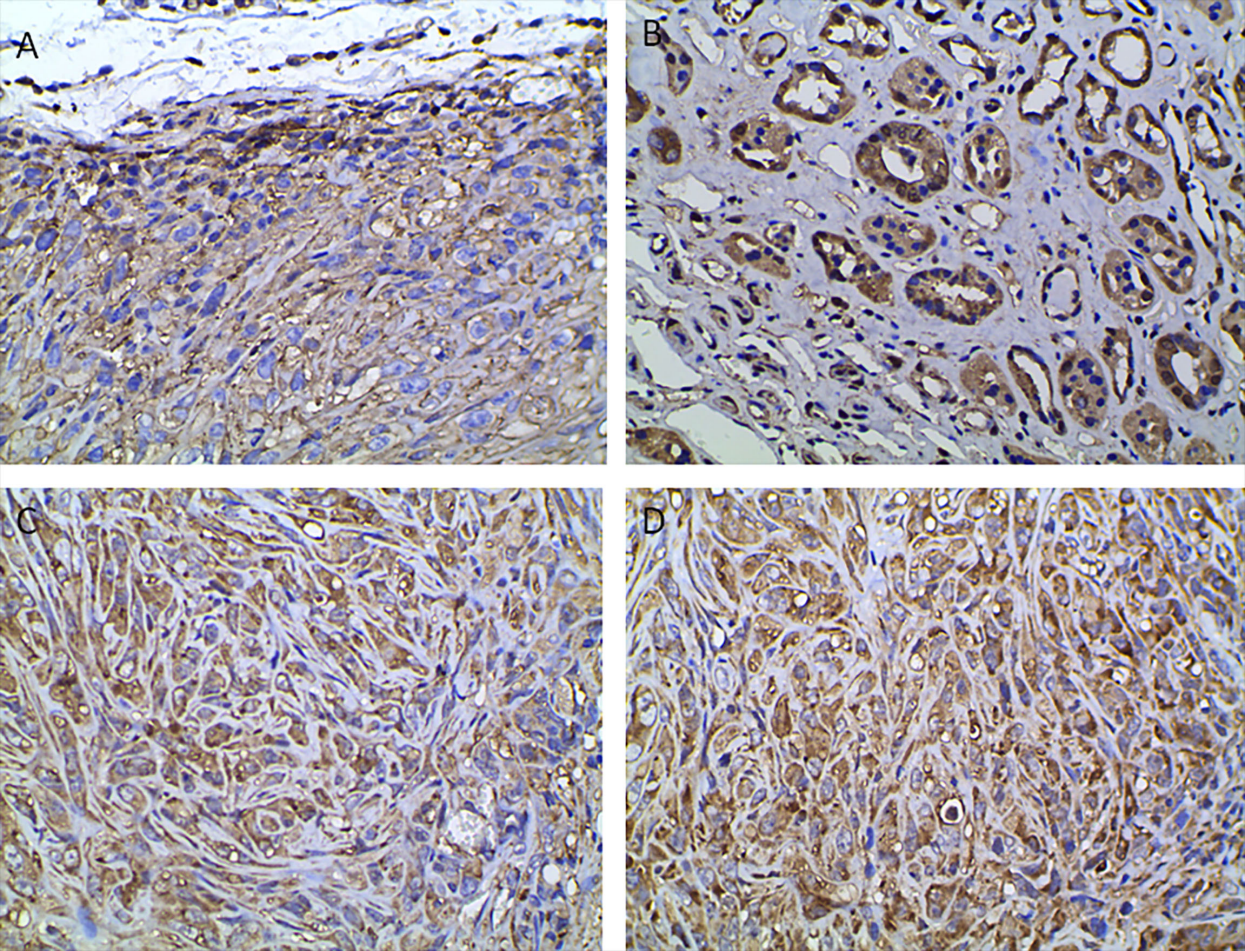
Immunohistochemical analysis indicating positive staining for enzymes associated with glucose metabolism and VHL. **(A)** GLUT1, **(B)** HK2, **(C)** LDHA, **(D)** VHL (400× magnification).

The high SUVmax and SUVmean values indicated that tumors with high PD-L1 levels had more active glycometabolism. Glucose metabolism activity in tumor cells is closely related to the expression of GLUT1, HK2, and LDHA (10, 11), which are enzymes involved in glucose metabolism (**Figures 4A–C**). Therefore, the correlations between the levels of enzymes related to glycometabolism and PD-L1 were assessed. We performed immunohistochemical staining for GLUT1, HK2, and LDHA (**Figures 4A–C**). The staining score was used to determine the correlation between the expression of GLUT1, HK2, and LDHA with PD-L1 levels. The results showed that that PD-L1 levels correlated weakly with LDHA levels. However, there was no significant association between GLUT1, HK2, or LDHA levels and PD-L1 levels (**Table 2**).

**Table 2:** Pearson correlation coefficients between SUVmax and the immunohistochemistry (IHC) staining scores for proteins associated with glucose metabolism and VHL in tumors.

Factor	PD-L1 expression	
	correlation coefficients	*P*-value
**GLUT-1 expression**	0.214	0.139
**HK-2 expression**	0.080	0.585
**LDHA expression**	0.280	0.035
**VHL expression**	0.347	0.011

*VHL* is a tumor suppressor gene, and mutations of *VHL* are closely related to the occurrence of renal cancer (12). We were interested in whether VHL levels in renal cancer correlated with PD-L1 levels (**Figure 4D**). The results showed that VHL levels in renal cancer correlated positively PD-L1 levels (r = 0.347, p = 0.011) (**Table 2**).

### Predictors of PD-L1 expression in CCRCC

PD-L1 expression was significantly associated with tumor size, renal venous invasion, lymph node metastasis, SUVmax, and SUVmean. Consequently, we further determined which parameters were independent predictors of PD-L1 using multivariate analysis, which showed that SUVmean and lymph node metastasis correlated significantly with PD-L1 levels in renal cell cancer (**Table 3**). Therefore, we used the lymph node metastasis and the PET SUVmean metabolic parameter to classify the PD-L1 positive rate of renal cancer lesions into a low-potential group (negative lymph node metastasis and SUVmean < 2.9), a moderate-potential group (negative lymph node metastasis and SUVmean ≥ 2.9, or positive lymph node metastasis and SUVmean < 2.9), and a high-potential group (positive lymph node metastasis and SUVmean ≥ 2.9). In these groups, the probability of PD-L1 expression was 20.59%, 29.41%, and 71.43%, respectively (P < 0.027; **Table 4**).

**Table 3:** Multivariate analyses of predictors of PD-L1 levels in patients with renal cancer patients.

Variable	Odds ratio	Confidence interval (95%)	*P-*value
**Lymph node metastasis**	4.613	1.034–20.577	0.045
**SUVmean**	1.332	1.027–1.728	0.031

**Table 4:** PD-L1 expression in the low-, moderate-, and high- potential group.

Probability	Total (n)	PD-L1 expression		*P-*value
		Low	High	
**Low**	34	79.41%	20.59%	< 0.027
**Moderate**	17	70.59%	29.41%	
**High**	7	28.57%	71.43%	

## Discussion

The expression level of PD-L1 affects the ability of PD1/PD-L1 immune checkpoint inhibitors (ICIs) to treat tumors. IHC assessment of PD-L1 levels is widely used as a biomarker to predict the response of tumors to ICIs (13). Although pathological diagnosis is the gold standard to evaluate PD-L1 levels, it is impossible to repeatedly examine biopsies of multiple lesions, and there is a need to develop non-invasive methods to assess PD-L1 levels in recurrent and metastatic disease.

To identify predictors of PD-L1 expression, the correlation between PD-L1 levels (represented by the IHC total score) and the patients’ clinical characteristics was assessed. The results showed that PD-L1 was expressed in a proportion of primary renal cancer cells. In addition, the PD-L1 level was associated with tumor size, renal venous invasion, and lymph node metastasis. Similar results were reported by Shen et al. (14). The level of PD-L1 in renal cell carcinoma (RCC) is associated with the lymph-gland transfer and necrosis rate, and in that study, PD-L1 levels were higher in patients with late tumor stage and poor prognosis (14). PD-L1 can be considered as a tumor promoter, because tumor progression is promoted by activation of the PD-1/PD-L1 pathway.

Research has demonstrated that PD-L1 expression correlates positively with SUVmax in colorectal cancer (15) and lung cancer (16, 17). Our study showed that SUVmax and SUVmean were significantly higher in primary renal cancer lesions with high levels of PD-L1 compared to those with low levels of PD-L1. PD-L1 levels correlated positively with SUVmax and SUVmean of renal cancer. As far as we know, this is the first report of an association between PD-L1 levels and 18F-FDG uptake in CCRCC. According to the areas under the SUVmax and SUVmean ROC curves, the uptake of 18F-FDG might predict PD-L1 levels.

According to multivariate analysis, SUVmean and lymph node metastasis correlated significantly with PD-L1 levels. Using the PET/CT imaging parameters, the patients were further categorized into groups according to their high, moderate, and low potential to be classified as PD-L1-positive. In the PD-L1-positive high potential group, up to 71.43% of tumors with lymph node metastasis and an SUVmean ≥ 2.9 were found to be PD-L1 positive.

Higher SUVmax and SUVmean values of renal cancers expressing PD-L1 indicates increased glycometabolism of the tumor cells (18). To date, a few studies have reported that tumor PD-L1 expression correlates with tumor glycometabolism regulation (19, 20). Therefore, we explored the relationships between PD-L1 levels and glucose metabolism related enzymes (GLUT1, HK2, and LDHA) in renal cancer, which showed that only LDHA levels correlated positively with PD-L1 levels. We speculated that this might be related to high expression of LDHA, which induces lactate production to activate the Tafazzin (TAZ)/PD-L1 signal pathway (21).

We were also interested in the correlation between VHL and PD-L1 levels. *VHL* is an important tumor suppressor gene, and *VHL* mutations induce renal carcinogenesis (22). Research has confirmed that the mutation status of *VHL* is related to PD-L1 expression (23). However, the correlation between the protein level of VHL and PD-L1 has not been reported in CCRCC. Herein, we found that PD-L1 levels correlated positively with VHL levels. It seems paradoxical that the levels oncogenic PD-L1 would correlate positively with the level of tumor suppressor VHL in renal cancer. The exact reason is unclear. We speculated that IHC staining can only detect the level of VHL protein, but cannot detect whether there is a mutation in the *VHL* gene. The mechanism underlying the correlation between VHL and PD-L1 levels deserves further study.

In conclusion, the study found that SUVmean and lymph node metastasis comprise independent factors that predict PD-L1 levels in CCRCC. Combining these two parameters can facilitate the non-invasive assessment of PD-L1 levels using PET/CT. In addition, we observed that PD-L1 levels correlated positively with LDHA and VHL levels in CCRCC. These results partially reveal the mechanism of CCRCC immune evasion and suggest new synergistic targets for immunotherapy.

## Data availability statement

The raw data supporting the conclusions of this article will be made available by the authors, without undue reservation.

## Ethics statement

All 58 patients met the criteria and informed consent was waived for the study, which was approved by the Institutional Review Board of shanghai 10th Hospital and was in accordance with the principles of the 2013 revision of the Declaration of Helsinki. The patients/participants provided their written informed consent to participate in this study. Written informed consent was obtained from the individual(s) for the publication of any potentially identifiable images or data included in this article.

## Author contributions

The study was designed by ZL and CM. YL, YH, and CM analyzed the data. YL and ZL wrote the original draft and edited it. All authors contributed to the article and approved the submitted version.

## Funding

This work was supported by the National Natural Science Foundation of China (82071964) and the Key Discipline Construction Project of Three**-**Year Action Plan (GWK-10.1-XK09).

## Conflict of interest

The authors declare that the research was conducted in the absence of any commercial or financial relationships that could be construed as a potential conflict of interest.

## Publisher’s note

All claims expressed in this article are solely those of the authors and do not necessarily represent those of their affiliated organizations, or those of the publisher, the editors and the reviewers. Any product that may be evaluated in this article, or claim that may be made by its manufacturer, is not guaranteed or endorsed by the publisher.
